# How could we suspect life‐threatening perinatal group A streptococcal infection?

**DOI:** 10.1111/jog.14497

**Published:** 2020-09-17

**Authors:** Tomohiro Arai, Yasushi Takai, Kouki Samejima, Shigetaka Matsunaga, Yoshihisa Ono, Hiroyuki Seki

**Affiliations:** ^1^ Center for Maternal, Fetal, and Neonatal Medicine, Saitama Medical Center Saitama Medical University Saitama Japan

**Keywords:** group A streptococcal infection, maternal sepsis, perinatal mortality

## Abstract

**Aim:**

Perinatal group A streptococcal infection is a rare but life‐threatening condition. Few reports have focused on its clinical characteristics and how to prevent deterioration. We report our experience with two antenatal fatal cases and reviewed 96 cases in the literature to assess the clinical characteristics of group A streptococcal infection.

**Methods:**

English‐language clinical reports of antenatal and postnatal group A streptococcal infection in 1974–2019 were retrieved and examined. Relationships between clinical characteristics and maternal outcomes were assessed.

**Results:**

Univariate analysis revealed that antenatal group A streptococcal infection was significantly associated with an age of ≤19 or ≥ 35 years, cesarean section, sore throat as an initial symptom, positive throat culture, maternal death and fetal death. Multivariate analysis revealed that antenatal onset (odds ratio = 7.922, 95% confidence interval = 1.297–48.374; *P* = 0.025) and a quick sepsis‐related organ‐failure assessment score (qSOFA; low blood pressure, high respiratory rate or altered mental status) of ≥2 (odds ratio = 6.166, 95% confidence interval = 1.066–35.670; *P* = 0.042) were significantly related to maternal death.

**Conclusion:**

Per our findings, antenatal group A streptococcal infection was significantly associated with maternal and fetal death. Further, the antenatal infection was revealed as a more critical risk factor. We suggest that the presence of any sign related to the qSOFA is a potential clue suspecting perinatal group A streptococcal infection in primary obstetric facilities.

## Introduction

Perinatal group A streptococcal (GAS) infection is a rare disease, occurring in six of every 100 000 births according to a North American report.^1^ The largest case study worldwide included only 67 cases.^2^ Even in the current age, in which hygienic conditions, antibiotics and intensive care settings have undergone significant advances, perinatal GAS infections can induce sudden septic shock (toxic shock syndrome^3^), resulting in the death of the mother and/or infants. Due to the rarity of the condition, detailed pathophysiology and its clinical characteristics have not been understood. Characteristics of vulnerable pregnant women, that is, important information for clinician, have also been unknown. We report two fatal cases, possibly due to GAS infection and analyzed the clinical features of perinatal GAS infection by reviewing the literature.

## Case Reports

### Case 1

*Patient*: A 26‐year‐old woman.

*Past obstetric history*: Gravida 2, para 1. Three years prior, the patient was transported to our hospital due to the rupture of membranes and labor with no prenatal care. An emergency cesarean section due to breech presentation was performed and a 2214 g healthy male was delivered.

*Past medical history*: Nothing remarkable.

*History of present illness*: At 13 weeks' gestation, the patient presented to a clinic with abdominal pain. She was diagnosed with a potential threat of miscarriage because of vaginal bleeding. Despite the offer of hospitalization, she refused and returned home. Six hours later, she experienced a rupture of the membranes and spontaneous abortion at home and thus returned to the clinic. The transvaginal ultrasound revealed a retained placenta; she decided to be admitted and undergo surgical treatment. However, she experienced sudden shortness of breath on admission, followed by altered mental status. Since her systolic blood pressure dropped to 60 mmHg, and peripheral capillary oxygen saturation (SpO2) also dropped to 60%, she was provided artificial ventilation. Despite the initial treatment, she went into cardiopulmonary arrest. She was transported to our tertiary medical center while being resuscitated. On arrival, she was still under cardiopulmonary arrest, and the resuscitation was continued. Her laboratory findings showed the following: white blood cell (WBC) count, 35 600 cells/μL; hemoglobin, 9.0 g/dL; platelet count, 113 000 cells/μL; D‐dimer, 32.0 μg/mL; fibrinogen, <70 mg/dL; blood urea nitrogen, 26.0 mg/dL; creatinine, 1.62 mg/dL; aspartate aminotransferase, 3993 U/L; alanine aminotransferase, 3041 U/L; C‐reactive protein, 8.4 mg/dL and procalcitonin, 9.5 ng/mL. GAS was later detected from a culture of a vaginal sample taken at this time. Transthoracic echocardiography revealed no right ventricular dilatation or pericardial effusion. Because she did not respond to resuscitation, percutaneous cardiopulmonary support was started. An enhanced computed tomography scan revealed no pulmonary embolism but diffuse infiltration in the lungs. She died 11 h and 36 min after the initial examination at the clinic.

### Case 2

*Patient*: A 37‐year‐old woman.

*Past obstetric history*: Gravida 1, para 0.

*Past medical history*: Acute hepatitis at 24 years of age.

*History of present illness*: The patient had regular prenatal check‐ups at a clinic. At 21 weeks' gestation, she presented with a fever of 38.0°C. Because she was suspected of having an influenza viral infection, she was prescribed oseltamivir and went home. Hypogastric pain appeared, and she returned to the clinic 6 h later. Upon arrival, her body temperature was 39°C, blood pressure was 91/62 mmHg and heart rate was 107 beats per minute. Intrauterine fetal death was diagnosed with transabdominal ultrasound. As the influenza point‐of‐care test result was negative and her WBC count elevated to 13 600/μL, intravenous flomoxef was initiated. Labor started after that, and she had a spontaneous abortion. The placenta was delivered simultaneously, revealing bloody amniotic fluid. Directly after the delivery, significant bleeding was noted, and the patient appeared to be drowsy. At this time, her blood pressure was 83/56 mmHg, heart rate was 124 beats per minute and postnatal hemorrhage had reached 1272 g. Therefore, she was transferred to a secondary medical center. Laboratory findings on arrival showed the following: WBC count, 32 800 cells/μL; hemoglobin, 8.1 g/dL; platelet count, 67 000 cells/μL; D‐dimer, 907.3 μg/mL; fibrinogen, <25 mg/dL; blood urea nitrogen, 31.0 mg/dL; creatinine, 2.28 mg/dL; aspartate aminotransferase, 146 U/L; alanine aminotransferase, 22 U/L and C‐reactive protein, 9.3 mg/dL. GAS was later identified from vaginal discharge taken at this hospital. She went into cardiopulmonary arrest, and cardiopulmonary resuscitation was provided. She restored spontaneous circulation 2 min later and was transferred to our tertiary medical center. She again went into cardiopulmonary arrest during the transfer. After arrival, she did not respond to continued attempts of cardiopulmonary resuscitation. She died 21 h and 46 min after the initial visit to the clinic.

Our two cases suspected of perinatal GAS infection had severe maternal outcomes with an astonishingly acute clinical course. Several studies have addressed the clinical characteristics of perinatal GAS infection. Of these, Hamilton *et al*. reported the largest number of cases (67 patients, including 10 antenatal) from the literature review.^2^ However, limited evidence is available on the clinical features indicative of perinatal GAS infection and, particularly, which patients require heightened attention. On the basis of the severe outcome of our cases, we hypothesized that antenatal GAS infection was associated with the outcome. To test this hypothesis, we compared the clinical characteristics of GAS infection between antenatal and postnatal onsets. We then assessed the clinical characteristics that affected the maternal outcome.

## Materials and Methods

### Data extraction

English‐language clinical reports describing antenatal and postnatal GAS infection between 1974 and 2019 were examined according to a PubMed database search using 'pregnancy' and 'group A *streptococcus*' as search terms.

Because data were incomplete in some of the published reports, we used the following definitions: Data were defined as a valid 'negative' finding when at least one finding, whether positive or negative, was reported for another item in the same category. Conversely, data were defined as 'missing' when no findings were reported for the category as a whole. For example, if abdominal pain was not reported as an initial symptom in a clinical report, abdominal pain was defined as 'negative' if at least one other initial symptom was reported, while abdominal pain was defined as 'missing' if no initial symptoms were reported. Missing data were not included in the analysis. Among the data collected, quick sepsis‐related organ failure assessment (qSOFA) score^4^ and CENTOR score (Cough absent, Exudate, Nodes, Temperature, young OR old modifier: a widely used diagnostic score for GAS pharyngitis)^5^ were dependent variables, whereas the other variables were independent variables. Cases in which vasopressor information was given were defined as 'systolic pressure <90 mmHg'. In the absence of actual numerical values, these terms were defined as follows: fever, body temperature ≥ 37.5°C; hypotension, systolic pressure < 90 mmHg; tachycardia, heart rate ≥ 100 beats per minute; tachypnea, respiratory rate > 20 breaths per minute; shock, systolic pressure < 90 mmHg and heart rate ≥ 100 beats per minute. Therefore, only the definition of the term 'fever' itself was not clearly achieved either as ≥38.0°C or ≥ 39.0°C. Cases in which the indicated clinical feature was not reported (i.e. missing) were not included in subsequent calculations.

### Statistical analysis

The above data were analyzed with IBM SPSS Statistics for Windows, version 23.0 (IBM Corp.). The following tests were used for comparison: Shapiro‐Wilks test for the distribution of variables, Student t‐test for normally distributed variables, Mann–Whitney U test for non‐normally distributed variables; two‐tailed Pearson's chi‐square test and one‐tailed Fisher's exact test for categorical variables; multivariate logistic regression analysis for regression analysis. The level of significance adopted was 5% (α = 0.05).

### Ethical approval

This study was approved by the ethical board at Saitama Medical Center (registration number: 2233).

## Results

We included a total of 98 patients reported over a long time period (45 years) (Table 1 ^6^, ^7^, ^8^, ^9^, ^10^, ^11^, ^12^, ^13^, ^14^, ^15^, ^16^, ^17^, ^18^, ^19^, ^20^, ^21^, ^22^, ^23^, ^24^, ^25^, ^26^, ^27^, ^28^, ^29^, ^30^, ^31^, ^32^, ^33^, ^34^, ^35^, ^36^, ^37^, ^38^, ^39^, ^40^, ^41^, ^42^, ^43^, ^44^, ^45^, ^46^, ^47^, ^48^, ^49^, ^50^, ^51^, ^52^, ^53^, ^54^, ^55^, ^56^, ^57^, ^58^, ^59^, ^60^, ^61^). Patient characteristics were compared between antenatal and postnatal GAS infection groups (Table 2). Of the 98 patients included, 25 were antenatal and 73 were postnatal infections. On univariate analysis, the antenatal GAS infection had a significant association with an age of ≤19 or ≥ 35 years, cesarean section, sore throat as an initial symptom, positive throat culture, maternal death and fetal death. In contrast, the postnatal GAS infection had a significant association with onset during hospitalization, abnormal vaginal discharge and any surgical interventions.

**Table 1:** Clinical cases of GAS infection reviewed in this study

Age	Onset of symptoms	Mode of delivery	Maternal outcome	Fetal outcome	Reference	Publication year
Antenatal onset (n = 25)						
Time of onset unknown (n = 1)						
22	NG	Vaginal delivery	Survived	Died	[6]	1993
Time of onset 1st trimester (n = 3)						
40	GW 7	Vaginal delivery	Survived	NG	[7]	1991
40	GW 10	Vaginal delivery	Died	Died	[8]	2015
**26**	**GW 13**	**Vaginal delivery**	**Died**	**Died**	**Case 1**	**2020**
Time of onset 2nd trimester (n = 5)						
32	GW 15	Vaginal delivery	Died	Died	[8]	2015
35	GW 18	NG	Died	Died	[8]	2015
**37**	**GW 21**	**Vaginal delivery**	**Died**	**Died**	**Case 2**	**2020**
21	GW 27	NG	Survived	NG	[9]	1996
19	GW 27	Vaginal delivery	Survived	Survived	[10]	2013
Time of onset 3rd trimester (n = 16)						
37	GW 28	Cesarean section	Died	NG	[11]	2001
24	GW 32	Cesarean section	Survived	Survived	[12]	2016
NG	GW 34	Cesarean section	Survived	Survived	[13]	1990
43	GW 34	Cesarean section	Died	Died	[14]	1996
29	GW 34	Vaginal delivery	Died	Died	[15]	1997
42	GW 34	Cesarean section	Died	Died	[15]	1997
31	GW 34	Cesarean section	Survived	Survived	[16]	2001
38	GW 34	Vaginal delivery	Died	Died	8	2015
35	GW 34	Vaginal delivery	Survived	Survived	[17]	2017
36	GW 35	NG	Died	Died	8	2015
32	GW 36	Cesarean section	Died	Died	[18]	1995
36	GW 36	Vaginal delivery	Died	Died	[19]	2001
36	GW 36	Vaginal delivery	Survived	NG	[20]	2007
16	GW 37	Vaginal delivery	Died	Died	[21]	2010
35	GW 37	NG	Died	Survived	[22]	2015
NG	GW 40	Vaginal delivery	Survived	Survived	[13]	1990
Postpartum onset (n = 73)						
Time of onset unknown (n = 12)						
29	NG	NG	Died	NG	[23]	1989
34	NG	NG	Survived	NG	[23]	1989
29	NG	Cesarean section	Survived	NG	[24]	2004
NG	NG	Vaginal delivery	Survived	Survived	[25]	2005
NG	NG	Vaginal delivery	Survived	Survived	[25]	2005
NG	NG	Vaginal delivery	Survived	Survived	[25]	2005
NG	NG	Vaginal delivery	Survived	Survived	[25]	2005
NG	NG	Vaginal delivery	Survived	Survived	[25]	2005
25	NG	NG	Survived	NG	[26]	2008
36	NG	NG	Survived	NG	[26]	2008
25	NG	NG	Survived	NG	[26]	2008
19	NG	NG	Survived	NG	[26]	2008
Time of onset 0–24 h (n = 21)						
NG	Immediately	Vaginal delivery	Survived	Survived	[13]	1990
31	Immediately	Vaginal delivery	Survived	Survived	[27]	1993
32	Immediately	Vaginal delivery	Survived	Survived	[30]	2018
19	25 min	Cesarean section	Died	Survived	[29]	1974
20	1 h	Vaginal delivery	Died	Survived	6	1994
33	A few hours	Vaginal delivery	Survived	NG	[22]	2017
27	8 h	Vaginal delivery	Survived	Survived	[30]	2008
24	12 h	Vaginal delivery	Survived	Survived	[31]	2005
27	12 h	Vaginal delivery	Survived	Survived	[32]	2011
32	16 h	Cesarean section	Survived	Survived	[19]	2001
33	18 h	Vaginal delivery	Survived	Survived	[19]	2001
28	20 h	Vaginal delivery	Died	Survived	[33]	1993
25	20 h	Vaginal delivery	Survived	Died	[27]	1993
21	24 h	Vaginal delivery	Died	Survived	[34]	1996
17	24 h	Vaginal delivery	Survived	Survived	[35]	1999
22	24 h	Vaginal delivery	Survived	NG	[19]	2001
NG	24 h	Vaginal delivery	Survived	Survived	[36]	2001
26	24 h	NG	Survived	NG	[26]	2008
34	24 h	Cesarean section	Died	Survived	[37]	2008
26	24 h	NG	Died	Survived	8	2015
28	24 h	Vaginal delivery	Survived	NG	[38]	2017
Time of onset 1–2 days (n = 14)						
28	29 h	Vaginal delivery	Survived	Survived	[39]	2008
39	30 h	Vaginal delivery	Died	Survived	[40]	2013
34	36 h	Vaginal delivery	Survived	NG	[22]	2017
27	2 days	Vaginal delivery	Survived	Survived	[41]	1992
36	2 days	Vaginal delivery	Survived	Survived	[42]	1995
29	2 days	Vaginal delivery	Survived	Survived	[43]	1996
26	2 days	Vaginal delivery	Survived	Survived	[44]	2003
29	2 days	Vaginal delivery	Survived	NG	[45]	2005
31	2 days	Vaginal delivery	Survived	Survived	[46]	2005
35	2 days	Cesarean section	Survived	Survived	[47]	2009
24	2 days	Vaginal delivery	Survived	NG	[10]	2013
27	2 days	Vaginal delivery	Survived	NG	[10]	2013
31	2 days	Vaginal delivery	Survived	NG	[48]	2013
28	2 days	Vaginal delivery	Survived	NG	[38]	2017
Time of onset 3–4 days (n = 14)						
36	3 days	Vaginal delivery	Survived	Survived	[49]	1993
NG	3 days	Vaginal delivery	Survived	Survived	[50]	2002
NG	3 days	Vaginal delivery	Died	Survived	[50]	2002
22	3 days	Vaginal delivery	Survived	NG	[51]	2009
29	3 days	Cesarean section	Died	Survived	[40]	2013
22	3 days	Vaginal delivery	Survived	NG	[38]	2017
37	3 days	Vaginal delivery	Survived	NG	[22]	2017
25	4 days	Vaginal delivery	Survived	Survived	[52]	1991
14	4 days	Vaginal delivery	Survived	Survived	[53]	1993
27	4 days	Vaginal delivery	Died	NG	9	1996
30	4 days	Vaginal delivery	Survived	NG	[54]	2001
NG	4 days	Vaginal delivery	Survived	Survived	[36]	2001
29	4 days	NG	Survived	NG	[26]	2008
37	4 days	Vaginal delivery	Survived	Survived	[55]	2015
Time of onset 5–7 days (n = 6)						
39	5 days	Vaginal delivery	Survived	Survived	[56]	2001
30	5 days	Vaginal delivery	Survived	NG	[57]	2008
35	5 days	Vaginal delivery	Died	NG	[22]	2017
37	6 days	Vaginal delivery	Survived	NG	9	1996
22	7 days	Vaginal delivery	Survived	Survived	[41]	1992
27	7 days	Vaginal delivery	Survived	NG	[10]	2013
Time of onset ≥8 days (n = 6)						
20	8 days	Vaginal delivery	Survived	NG	[19]	2001
26	13 days	Cesarean section	Survived	NG	[58]	2003
23	14 days	Vaginal delivery	Survived	Survived	[59]	1990
36	22 days	NG	Survived	NG	[26]	2008
23	3 weeks	Cesarean section	Survived	Survived	[60]	1984
27	5 weeks	Vaginal delivery	Survived	Survived	[61]	2005

Bold characters indicate cases that we experienced.

GAS, group A streptococcal; GW, gestational weeks; NG, not given.

**Table 2:** Clinical characteristics of perinatal GAS infection

Characteristics of GAS infection	No. (%) of patients with the indicated feature^†^ ^,^ ^‡^		*P*‐value
	Antenatal	Postnatal	
	(n = 25)	(n = 73)	
Age, ≤19 or ≥35 years	15 / 23 (65.2)	15 / 63 (23.8)	0.000^*^
Multiparous	13 / 17 (76.5)	35 / 45 (77.8)	0.580
Onset during hospitalization	1 / 25 (4.0)	26 / 65 (40.0)	0.001^*^
Mode of delivery			
Vaginal delivery	13 / 20 (65.0)	54 / 62 (87.1)	0.034^**^
Cesarean section	7 / 20 (35.0)	8 / 62 (12.9)	
Initial symptoms			
Fever, chills, full‐like symptoms	16 / 24 (66.7)	45 / 60 (75.0)	0.439
Sore throat with or without other upper respiratory symptoms	7 / 24 (29.2)	5 / 60 (8.3)	0.020^**^
Nausea, vomiting, diarrhea	6 / 24 (25.0)	8 / 60 (13.3)	0.165
Abdominal pain	8 / 24 (33.3)	34 / 60 (56.7)	0.053
Clinical features			
Fever (≥38.0°C)	11 / 19 (57.9)	26 / 57 (45.6)	0.354
Hypotension (systolic pressure ≤ 90 mmHg)	10 / 19 (52.6)	26 / 57 (45.6)	0.596
Tachycardia (≥100 beats per min)	11 / 19 (57.9)	31 / 57 (54.4)	0.790
Leukocytosis (WBC count >11 000/mm^3^)	4 / 19 (21.1)	20 / 57 (35.1)	0.254
Uterine tenderness	3 / 19 (15.8)	17 / 57 (29.8)	0.229
Abnormal vaginal discharge	2 / 19 (10.5)	20 / 57 (35.1)	0.041^*^
Erythema	3 / 19 (15.8)	15 / 57 (26.3)	0.273
Extremity pain	2 / 19 (10.5)	13 / 57 (22.8)	0.207
Sore throat+CENTOR, ≥2	4 / 11 (36.4)	4 / 6 (66.7)	0.247
qSOFA score, ≥2	6 / 15 (40.0)	15 / 59 (25.4)	0.210
Pharmacological interventions			
Antibiotic treatment	19 / 21 (90.5)	67 /68 (98.5)	0.137
Immunoglobulin (IVIG)	4 / 21 (19.0)	4 / 68 (5.9)	0.085
Surgical interventions			
Debridement, drainage and/or amputation of extremities	1 / 21 (4.8)	11 / 43 (25.6)	0.041^**^
Exploratory surgery (laparotomy)	5 / 21 (23.8)	26 / 43 (60.5)	0.006^*^
Hysterectomy	4 / 21 (19.0)	20 / 43 (46.5)	0.033^*^
Surgical findings			
Ascites or pus in the peritoneal cavity (included peritonitis)	3 / 5 (60.0)	8 / 12 (66.7)	0.793
Necrosis, inflammation, or exudate present in the uterus, ovaries and/or fallopian tubes	4 / 6 (66.7)	20 / 22 (90.9)	0.191
Normal placenta, uterus and/or pelvic organs	2 / 6 (33.3)	2 / 22 (9.1)	0.191
Bacterial sources			
Urine	0 / 22 (0.0)	9 / 72 (12.5)	0.080
Cervix, vagina, lochia	9 / 22 (40.9)	40 / 72 (55.6)	0.229
Peritoneum (ascites)	2 / 22 (9.1)	11 / 72 (15.3)	0.368
Oropharynx or respiratory system	5 / 22 (22.7)	4 / 72 (5.6)	0.030^**^
Blood	16 / 22 (72.7)	45 / 72 (62.5)	0.379
CNS system	1 / 22 (4.5)	1 / 72 (1.4)	0.415
Maternal outcome			
Survived	10 / 25 (40.0)	61 / 73 (83.6)	0.000^*^
Died	15 / 25 (60.0)	12 / 73 (16.4)	
Fetal outcome			
Survived	7 / 25 (33.3)	41 / 43 (95.3)	0.000^*^
Died	14 / 25 (66.7)	2 / 43 (4.7)	

^*^ A two‐tailed paired Pearson's chi‐square test with α = 0.05, yielded a statistically significant *P*‐value. Actual values were noted in the table.

^**^ A one‐tailed paired Fisher's exact test with α = 0.05 yielded a statistically significant *P*‐value. Actual values were noted in the table.

^†^ Data were extracted as follows: A valid negative finding was recorded when at least one other positive or negative finding was reported for a specific category. Data were recorded as missing when there were no findings reported for the category. Cases in which a vasopressor was administered were recorded as having a 'systolic pressure <90 mmHg'. When numerical values were reported, the terms were defined as follows: *fever*, body temperature ≥ 37.5°C; *hypotension*, systolic pressure < 90 mmHg; *tachycardia*, heart rate ≥ 100 beats per minute; *tachypnea*, respiratory rate > 20 breaths per minute; *shock*, systolic pressure < 90 mmHg and heart rate ≥ 100 beats per minute. Thus 'fever' could not be stratified as either ≥38.0°C or ≥ 39.0°C.

^‡^ Missing data were excluded from the analysis. They were also excluded from each denominators.

Cases were described in Table 1.

CNS, central nervous system; GAS, group A streptococcal; IVIG, intravenous immunoglobulin; qSOFA, quick sequential organ failure assessment; CENTOR, cough absent, exudate, nodes, temperature, young OR old modifier; WBC, white blood cell.

Univariate analysis was used to test the relationship between the maternal outcome and several clinical characteristics assumed to be potential risk factors (Table 3). Five clinical characteristics: antenatal onset, age of ≤19 or ≥ 35 years, cesarean section, qSOFA score of ≥2 and no surgical interventions were significantly associated with maternal death on univariate analysis. Next, multivariate analysis was performed with the abovementioned five clinical characteristics (Table 4). Antenatal onset and a qSOFA score of ≥2 were significantly and independently associated with maternal death (odds ratio [OR] = 7.922, 95% confidence interval [CI] = 1.297–48.374; *P* = 0.025, and OR = 6.166, 95% CI = 1.066–35.670; *P* = 0.042, respectively).

**Table 3:** Clinical risk factors for perinatal GAS infection (univariate analysis)

Characteristics	Maternal outcome		*P*‐value
	Survived (n = 71)	Died (n = 27)	
Antenatal onset	10 (14.1)	15 (55.6)	0.000^*^
Age, ≤19 or ≥35 years	16 (26.7)	14 (53.8)	0.015^*^
Multiparous	33 (76.7)	15 (78.9)	0.564
Cesarean section	8 (13.1)	7 (33.3)	0.045^**^
Onset during hospitalization	20 (31.3)	7 (26.9)	0.685
Sore throat + CENTOR, ≥2	6 (60.0)	2 (28.6)	0.218
qSOFA score, ≥2	11 (19.3)	10 (58.8)	0.003^**^
No surgical intervention	14 (28.6)	12 (63.2)	0.008^*^
Positive blood culture	43 (62.3)	18 (72.0)	0.385

^*^ A two‐tailed paired Pearson's chi‐square test (α = 0.05) performed on this data yielded a statistically significant *P*‐value. Actual values were noted in the table.

^**^ A one‐tailed paired Fisher's exact test (α = 0.05) performed on this data yielded a statistically significant *P*‐value. Actual values were noted in the table.

Numerical values were defined as n (%), and cases in which the indicated feature was not reported were not included in calculations.

CENTOR, cough absent, exudate, nodes, temperature, young OR old modifier; GAS, group A streptococcal; qSOFA, quick sequential organ failure assessment.

**Table 4:** Multivariate analysis evaluating risk factors for maternal mortality caused by perinatal GAS infection

Characteristics	Maternal outcome		OR	95% CI	*P*
	Survived (n = 71)	Died (n = 27)			
Antenatal onset	10 (14.1)	15 (55.6)	7.922	1.297–48.374	0.025^*^
qSOFA score, ≥2	11 (19.3)	10 (58.8)	6.166	1.066–35.670	0.042^*^
No surgical intervention	14 (28.6)	12 (63.2)	NG	NG	NG
Age, ≤19 or ≥35 years	16 (26.7)	14 (53.8)	NG	NG	NG
Cesarean section	8 (13.1)	7 (33.3)	NG	NG	NG

^*^ Logistic regression analysis (α = 0.05) identified that antenatal onset and a qSOFA score of ≥2 were to be independent risk factors associated with maternal death in perinatal GAS infection.

Numerical values were defined as n (%), and cases in which the indicated feature was not reported were not included in calculations.

CI, confidence interval; GAS, group A streptococcal; NG, not given.

## Discussion

Antenatal onset and a qSOFA score of ≥2 were significantly associated with maternal death in our study. In the literature, Hamilton *et al*. reported the largest number of cases of perinatal GAS infections (67 patients, including 10 with antenatal infection) and noted that GAS infection during the third trimester was associated with maternal and fetal death,^2^ whereas we reported a total of 98 patients, including 25 with antenatal infection. Of particular interest is the comparison between clinical characteristics and maternal outcome by multivariate analysis, which was not attempted in previous reports. Furthermore, few reports have included CENTOR criteria, defined as Cough absent, Exudate, Nodes, Temperature, young OR old modifier, and the qSOFA score as a clinical characteristic. Only Tanaka *et al*. examined the qSOFA score in maternal death cases.^62^

Why was antenatal GAS infection associated with poor maternal outcomes? Compared with postnatal cases, antenatal infections had initial symptoms outside of the hospital and the initiation of treatment might have been delayed. Generally, potentially invasive surgical treatment often makes physicians hesitant to operate upon pregnant patients.

Second, we assume there may be several types of perinatal GAS infection depending on the infection route, as well as the initial infection site. However, as is mentioned in previous article,^63^ it is difficult to elucidate the route of infection of GAS, and we were not able to extract valid data from articles.

We next investigated potential indicators of risk that could be used to suspect a perinatal GAS infection in pregnant women. Although the CENTOR criteria is a diagnostic criterion for GAS pharyngitis and the qSOFA score is that for sepsis, we assumed that these criteria can be used for the detection of perinatal GAS infection. However, because of the limited data availability, we were not able to examine the sensitivity in this study. In antenatal cases, all the criteria in the qSOFA score (systolic pressure < 100 mmHg, respiratory rate > 22 breaths per minute and altered mental status) were available in 15 cases. Of these, 13 cases (86.7%) satisfied one or more criteria in qSOFA. Thus, we suggest that medical resources should be provided earlier to pregnant women who feel ill, and medical providers should triage using the qSOFA score. If a patient is positive for any of the qSOFA criteria, she should be immediately referred to a critical care medical center. Further study should be done because of the limited number of cases in this study.

There were some other limitations to our study that should be considered. Because patients from a wide time period were included, patients from a more recent chronological timeframe may have experienced better outcomes than those from an earlier chronological period. However, the maternal outcome was not related to the published year of the case report (*P* = 0.363, Mann Whitney U test). Since most cases did not fully describe all the data we required (respiratory rate, in particular) for the study, we were not able to exclude the possibility of selection bias. In addition, our two cases are not definite cases, but probable cases according to the definition of Streptococcal toxic shock syndrome.^3^ Likewise, our analyses included the other 28 probable cases extracted from past articles.

In conclusion, antenatal GAS infection is more critical than postnatal GAS infection, and a qSOFA score of ≥2 is also potentially critical. We suggest that the presence of any of the qSOFA signs may represent a useful clinical marker for antenatal GAS infection among patients presenting at primary obstetric facilities.

## Disclosure

The authors report no conflict of interest.

## References

[1] Chuang I , Van Beneden C , Beall B *et al* Population‐based surveillance for postpartum invasive group a streptococcus infections, 1995–2000. Clin Infect Dis 2002; 35: 665–670. 12203162 10.1086/342062

[2] Hamilton SM , Stevens DL , Bryant AE . Pregnancy‐related group a streptococcal infections: Temporal relationships between bacterial acquisition, infection onset, clinical findings, and outcome. Clin Infect Dis 2013; 57: 870–876. 23645851 10.1093/cid/cit282 PMC3749745

[3] Lappin E , Ferguson AJ . Gram‐positive toxic shock syndromes. Lancet Infect Dis 2009; 9: 281–290. 19393958 10.1016/S1473-3099(09)70066-0

[4] Singer M , Deutschman CS , Seymour CW *et al* The third international consensus definitions for sepsis and septic shock (Sepsis‐3). JAMA 2016; 315: 801–810. 26903338 10.1001/jama.2016.0287 PMC4968574

[5] McIsaac WJ , White D , Tannenbaum D , Low DE . A clinical score to reduce unnecessary antibiotic use in patients with sore throat. CMAJ 1998; 158: 75–83. 9475915 PMC1228750

[6] Nathan L , Peters MT , Ahmed AM , Leveno KJ . The return of life‐threatening puerperal sepsis caused by group a streptococci. Am J Obstet Gynecol 1993; 169: 571–572. 8372865 10.1016/0002-9378(93)90623-q

[7] Dotters DJ , Katz VL . Streptococcal toxic shock associated with septic abortion. Obstet Gynecol 1991; 78: 549–551. 1870820

[8] Hasegawa J , Sekizawa A , Yoshimatsu J *et al* Cases of death due to serious group a streptococcal toxic shock syndrome in pregnant females in Japan. Arch Gynecol Obstet 2015; 291: 5–7. 25194311 10.1007/s00404-014-3440-0

[9] McHenry CR , Azar T , Ramahi AJ , Collins PL . Monomicrobial necrotizing fasciitis complicating pregnancy and puerperium. Obstet Gynecol 1996; 87: 823–826. 8677102

[10] Busowski MT , Lee M , Busowski JD , Akhter K , Wallace MR . Puerperal group a streptococcal infections: A case series and discussion. Case Rep Med 2013; 2013: 751329. 23710192 10.1155/2013/751329 PMC3638550

[11] Hirose Y , Shibuya H , Okazaki E *et al* Toxic shock‐like syndrome with flu‐like prodrome: A possible role of 'enhancing tissue focus' for streptococcal toxic shock. J Infect 2001; 42: 195–200. 11545551 10.1053/jinf.2001.0820

[12] Imaeda T , Nakada TA , Abe R , Tateishi Y , Oda S . Veno‐arterial extracorporeal membrane oxygenation for *Streptococcus pyogenes* toxic shock syndrome in pregnancy. J Artif Organs 2016; 19: 200–203. 26758056 10.1007/s10047-015-0884-3

[13] Belfrage E , Anzén B , Jörbeck H , Sterner G , Marland M . Streptococcal infections in late pregnancy and labor. Scand J Infect Dis Suppl 1990; 71: 79–85. 2287920

[14] Ichiyama S , Nakashima K , Shimokata K *et al* Transmission of *Streptococcus pyogenes* causing toxic shock‐like syndrome among family members and confirmation by DNA macrorestriction analysis. J Infect Dis 1997; 175: 723–726. 9041354 10.1093/infdis/175.3.723

[15] Ooe K , Udagawa H . A new type of fulminant group a streptococcal infection in obstetric patients: Report of two cases. Hum Pathol 1997; 28: 509–512. 9104954 10.1016/s0046-8177(97)90043-5

[16] Crum NF , Chun HM , Gaylord TG , Hale BR . Group a streptococcal toxic shock syndrome developing in the third trimester of pregnancy. Infect Dis Obstet Gynecol 2002; 10: 209–216. 12648315 10.1155/S1064744902000248 PMC1784620

[17] Alhousseini A , Layne ME , Gonik B , Bryant D , Patwardhan S , Patwardhan M . Successful continuation of pregnancy after treatment of group a streptococci sepsis. Obstet Gynecol 2017; 129: 907–910. 28383376 10.1097/AOG.0000000000001967

[18] Morgan PJ . Maternal death following epidural anaesthesia for caesarean section delivery in a patient with unsuspected sepsis. Can J Anaesth 1995; 42: 330–334. 7788829 10.1007/BF03010709

[19] Barnham MR , Weightman NC . Bacteraemic *Streptococcus pyogenes* infection in the peri‐partum period: Now a rare disease and prior carriage by the patient may be important. J Infect 2001; 43: 173–176. 11798254 10.1053/jinf.2001.0887

[20] Palep‐Singh M , Jayaprakasan J , Hopkisson JF . Peripartum group a streptococcal sepsis: A case report. J Reprod Med 2007; 52: 977–978. 17977181

[21] Sugiyama T , Kobayashi T , Nagao K , Hatada T , Wada H , Sagawa N . Group A streptococcal toxic shock syndrome with extremely aggressive course in the third trimester. J Obstet Gynaecol Res 2010; 36: 852–855. 20666956 10.1111/j.1447-0756.2010.01259.x

[22] Gustafson LW , Blaakær J , Helmig RB . Group A streptococci infection. A systematic clinical review exemplified by cases from an obstetric department. Eur J Obstet Gynecol Reprod Biol 2017; 215: 33–40. 28600919 10.1016/j.ejogrb.2017.05.020

[23] Stevens DL , Tanner MH , Winship J *et al* Severe group A streptococcal infections associated with a toxic shock‐like syndrome and scarlet fever toxin A. N Engl J Med 1989; 321: 1–7. 2659990 10.1056/NEJM198907063210101

[24] Okumura K , Schroff R , Campbell R , Nishioka L , Elster E . Group A streptococcal puerperal sepsis with retroperitoneal involvement developing in a late postpartum woman: Case report. Am Surg 2004; 70: 730–732. 15328810

[25] Raymond J , Schlegel L , Garnier F , Bouvet A . Molecular characterization of *Streptococcus pyogenes* isolates to investigate an outbreak of puerperal sepsis. Infect Control Hosp Epidemiol 2005; 26: 455–461. 15954483 10.1086/502567

[26] Aronoff DM , Mulla ZD . Postpartum invasive group A streptococcal disease in the modern era. Infect Dis Obstet Gynecol 2008; 2008: 796892 10.1155/2008/796892. 19125207 PMC2612756

[27] Panaro NR , Lutwick LI , Chapnick EK . Intrapartum transmission of group a streptococcus. Clin Infect Dis 1993; 17: 79–81. 8353251 10.1093/clinids/17.1.79

[28] Nguyen M , Bendi VS , Guduru M *et al* Postpartum invasive group A streptococcus infection: Case report and mini‐review. Cureus 2018; 10: e3184. 30364777 10.7759/cureus.3184 PMC6199147

[29] Jewett JF . Post‐cesarean sepsis. N Engl J Med 1974; 291: 1032–1033. 4415143 10.1056/NEJM197411072911913

[30] Slowey MJ , Piacquadio KM . Generalized Shwartzman reaction caused by group a beta‐hemolytic streptococcus, resulting in maternal death. A case report. J Reprod Med 1994; 39: 553–556. 7966050

[31] Castagnola DE , Hoffman MK , Carlson J , Flynn C . Necrotizing cervical and uterine infection in the postpartum period caused by group a streptococcus. Obstet Gynecol 2008; 111: 533–535. 18239012 10.1097/01.AOG.0000284453.41550.66

[32] Lee VH , Sulis C , Sayegh RA . Puerperal Group A Streptococcus infection: A case report. J Reprod Med 2005; 50: 621–623. 16220770

[33] Dehaene I , Loccufier A , Temmerman M , de Keersmaecker B , de Baene L . Creatine kinase as an indicator for hysterectomy in postpartum endomyometritis due to group A streptococci: A hypothesis illustrated by a case report. Gynecol Obstet Invest 2012; 73: 82–88. 22178984 10.1159/000333233

[34] Noronha S , Yue CT , Sekosan M . Puerperal group a beta‐hemolytic streptococcal toxic shock‐like syndrome. Obstet Gynecol 1996; 88: 728. 8841274 10.1016/0029-7844(96)00214-1

[35] Gergis H , Barik S , Lim K , Porter W . Life‐threatening puerperal infection with group a streptococcus. J R Soc Med 1999; 92: 412–413. 10656011 10.1177/014107689909200811 PMC1297321

[36] Stefonek KR , Maerz LL , Nielsen MP , Besser RE , Cieslak PR . Group A streptococcal puerperal sepsis preceded by positive surveillance cultures. Obstet Gynecol 2001; 98: 846–848. 11704180 10.1016/s0029-7844(01)01611-8

[37] González Castro A , Rodriguez‐Borregan JC , Obeso T , Castellanos A , Perez‐Ceballos A , Sesmero JR . Necrotizing fasciitis after cesarean section. Arch Gynecol Obstet 2008; 277: 579–581. 18004578 10.1007/s00404-007-0509-z

[38] Cooper JD , Cooper SR , Wolk D *et al* Postpartum *Streptococcus pyogenes* outbreak in the labor and delivery unit of a quaternary referral center: A case series and review of the literature. Clin Microbiol Newsl 2017; 39: 11–15.

[39] Lurie S , Vaknine H , Izakson A , Levy T , Sadan O , Golan A . Group A Streptococcus causing a life‐threatening postpartum necrotizing myometritis: A case report. J Obstet Gynaecol Res 2008; 34: 645–648. 18840172 10.1111/j.1447-0756.2008.00900.x

[40] Turner CE , Dryden M , Holden MT *et al* Molecular analysis of an outbreak of lethal postpartum sepsis caused by *Streptococcus pyogenes* . J Clin Microbiol 2013; 51: 2089–2095. 23616448 10.1128/JCM.00679-13 PMC3697669

[41] Silver RM , Heddleston LN , McGregor JA , Gibbs RS . Life‐threatening puerperal infection due to group a streptococci. Obstet Gynecol 1992; 79: 894–896. 1565401

[42] Rowan JA , North RA . Necrotizing fasciitis in the puerperium. Am J Obstet Gynecol 1995; 173: 241–242. 7631696 10.1016/0002-9378(95)90205-8

[43] Jorup‐Rönström C , Hofling M , Lundberg C , Holm S . Streptococcal toxic shock syndrome in a postpartum woman. Case report and review of the literature. Infection 1996; 24: 164–167. 8740114 10.1007/BF01713330

[44] Golden S . Group a streptococcus and streptococcal toxic shock syndrome: A postpartum case report. J Midwifery Womens Health 2003; 48: 357–359. 14526350 10.1016/s1526-9523(03)00284-8

[45] Torda A . Postpartum toxic shock syndrome associated with multiple splenic infarcts. Med J Aust 2005; 182: 93. 10.5694/j.1326-5377.2005.tb06588.x 15651973

[46] Yagi H , Fukushima K , Satoh S , Nakashima Y , Nozaki M , Nakano H . Postpartum retroperitoneal fasciitis: A case report and review of literature. Am J Perinatol 2005; 22: 109–113. 15731991 10.1055/s-2005-861573

[47] de Moya MA , del Carmen MG , Allain RM , Hirschberg RE , Shepard JAO , Kradin RL . Case records of the Massachusetts General Hospital. Case 33‐2009. A 35‐year‐old woman with fever, abdominal pain, and hypotension after cesarean section. N Engl J Med 2009; 361: 1689–1697. 19846855 10.1056/NEJMcpc0900646

[48] Park YS , Owen AM , Adno AM , Marry J . Pyogenic pacroiliitis due to Group A Streptococcus following uncomplicated pregnancy and vaginal delivery. Case Rep Obstet Gynecol 2013; 2013: 981474. 24396619 10.1155/2013/981474 PMC3874360

[49] Keogh J , MacDonald D , Kelehan P . Septic pelvic thrombophlebitis: An unusual treatable postpartum complication. Aust N Z J Obstet Gynaecol 1993; 33: 204–207. 8216128 10.1111/j.1479-828x.1993.tb02395.x

[50] Schummer W , Schummer C . Two cases of delayed diagnosis of postpartal streptococcal toxic shock syndrome. Infect Dis Obstet Gynecol 2002; 10: 217–222. 12648316 10.1155/S106474490200025X PMC1784622

[51] Daif JL , Levie M , Chudnoff S , Kaiser B , Shahabi S . Group a streptococcus causing necrotizing fasciitis and toxic shock syndrome after medical termination of pregnancy. Obstet Gynecol 2009; 113: 504–506. 19155935 10.1097/AOG.0b013e3181834626

[52] Martens PR , Mullie A , Goessens L . A near‐fatal case of puerperal sepsis. Anaesth Intensive Care 1991; 19: 108–110. 2012267 10.1177/0310057X9101900122

[53] Snabes MC , Martens MG . A severe puerperal group A streptococcal infection causing a toxic shock‐like syndrome. Int J Gynaecol Obstet 1993; 40: 245–248. 8096477 10.1016/0020-7292(93)90838-n

[54] Christie JH , Keay SD , Morgan M , Storley R . Puerperal sepsis: A disease of the past? BJOG 2001; 108: 127 10.1111/j.1471-0528.2001.00046.x. 11212990

[55] Keller NA , Guan X , Wiczulis A , Burcher P . Unexplained persistent postpartum palpitations and tachycardia due to Group A Streptococcus. BMC Res Notes 2015; 8: 731 10.1186/s13104-015-1739-y. 26619910 PMC4666222

[56] Gourlay M , Gutierrez C , Chong A , Robertson R . Group A streptococcal sepsis and ovarian vein thrombosis after an uncomplicated vaginal delivery. J Am Board Fam Pract 2001; 14: 375–380. 11572543

[57] Feigenberg T , Sela HY , Applbaum YH , Mankuta D . Puerperal widespread pyomyositis after group A streptococcal toxic shock syndrome. Isr Med Assoc J 2008; 10: 483–484. 18669156

[58] Synnestvedt M , Müller F , Gaustad P , Bucher A . Recurrent group a streptococcal genital infection after puerperal sepsis. Scand J Infect Dis 2003; 35: 509–510. 14514155 10.1080/00365540310013261

[59] Whitted RW , Yeomans ER , Hankins GD . Group A beta‐hemolytic streptococcus as a cause of toxic shock syndrome. A case report. J Reprod Med 1990; 35: 558–560. 2191134

[60] Quinney RA , Ali MR , Thomas MG , Lang SD . Post‐partum streptococcal meningitis and septicaemia. N Z Med J 1984; 97: 702–703. 6384842

[61] Griffiths AN , Sudhahar AA , Ashraf M . Neonatal necrotising fasciitis and late maternal pelvic abscess formation. A late complication of group a streptococcus. J Obstet Gynaecol 2005; 25: 197–198. 15814407 10.1080/01443610500051320

[62] Tanaka H , Katsuragi S , Hasegawa J *et al* The most common causative bacteria in maternal sepsis‐related deaths in Japan were group A Streptococcus: A nationwide survey. J Infect Chemother 2019; 25: 41–44. 30377069 10.1016/j.jiac.2018.10.004

[63] Meleney FL . Hemolytic streptococcus gangrene. Arch Surg 1924; 9: 317–364.

